# Cellular Mechanisms Underlying B Cell Abnormalities in Patients With Gain-of-Function Mutations in the *PIK3CD* Gene

**DOI:** 10.3389/fimmu.2022.890073

**Published:** 2022-06-21

**Authors:** Wenjie Wang, Qing Min, Nannan Lai, Krisztian Csomos, Ying Wang, Luyao Liu, Xin Meng, Jinqiao Sun, Jia Hou, Wenjing Ying, Qinhua Zhou, Bijun Sun, Xiaoying Hui, Boglarka Ujhazi, Sumai Gordon, David Buchbinder, Catharina Schuetz, Manish Butte, Jolan E. Walter, Xiaochuan Wang, Ji-Yang Wang

**Affiliations:** ^1^ Department of Clinical Immunology, Children’s Hospital of Fudan University, National Children’s Medical Center, Shanghai, China; ^2^ Key Laboratory of Whole-Period Monitoring and Precise Intervention of Digestive Cancer, Shanghai Municipal Health Commission (SMHC), Minhang Hospital, Fudan University, Shanghai, China; ^3^ Division of Pediatric Allergy/Immunology and Jeffrey Modell Diagnostic and Research Center, University of South Florida and Johns Hopkins All Children’s Hospital, St. Petersburg, FL, United States; ^4^ Department of Immunology, School of Basic Medical Sciences, Fudan University, Shanghai, China; ^5^ Division of Hematology, Children’s Hospital of Orange Country (CHOC), Irvine, CA, United States; ^6^ Department of Pediatrics, Medizinische Fakultät Carl Gustav Carus, Technische Universität Dresden, Dresden, Germany; ^7^ Division of Immunology, Allergy, and Rheumatology, Department of Pediatrics and Jeffrey Modell Diagnostic and Research Center, University of California, Los Angeles, Los Angeles, CA, United States; ^8^ Massachusetts General Hospital, Boston, MA, United States; ^9^ Shanghai Institute of Infectious Disease and Biosecurity, Shanghai, China; ^10^ Department of Microbiology and Immunology, College of Basic Medical Sciences, Zhengzhou University, Zhengzhou, China; ^11^ Division of Biochemistry, Faculty of Pharmacy and Graduate School of Pharmaceutical Science, Keio University, Tokyo, Japan

**Keywords:** activated PI3Kδ syndrome (APDS), B cell survival and activation, CD27^-^IgD^-^ double-negative B cells, Ig gene class switch recombination, plasma cell differentiation

## Abstract

**Background:**

Activated phosphoinositide 3 kinase (PI3K) -delta syndrome (APDS) is an inborn error of immunity with variable clinical phenotype of immunodeficiency and immune dysregulation and caused by gain-of-function mutations in *PIK3CD*. The hallmark of immune phenotype is increased proportions of transitional B cells and plasmablasts (PB), progressive B cell loss, and elevated levels of serum IgM.

**Objective:**

To explore unique B cell subsets and the pathomechanisms driving B cell dysregulation beyond the transitional B cell stage in APDS.

**Methods:**

Clinical and immunological data was collected from 24 patients with APDS. In five cases, we performed an in-depth analysis of B cell phenotypes and cultured purified naïve B cells to evaluate their survival, activation, Ig gene class switch recombination (CSR), PB differentiation and antibody secretion. We also analyzed PB differentiation capacity of sorted CD27^-^IgD^-^ double-negative B (DNB) cells.

**Results:**

The patients had increased B cell sizes and higher proportions of IgM^+^ DNB cells than healthy controls (HC). Their naïve B cells exhibited increased death, impaired CSR but relatively normal PB differentiation. Upon stimulation, patient’s DNB cells secreted a similar level of IgG but a higher level of IgM than DNB cells from HC. Targeted therapy of PI3K inhibition partially restored B cell phenotypes.

**Conclusions:**

The present study suggests additional mechanistic insight into B cell pathology of APDS: (1) decreased peripheral B cell numbers may be due to the increased death of naïve B cells; (2) larger B cell sizes and expanded DNB population suggest enhanced activation and differentiation of naïve B cells into DNB cells; (3) the impaired CSR yet normal PB differentiation can predominantly generate IgM-secreting cells, resulting in elevated IgM levels.

## Introduction

Naïve mature B cells are normally in a resting state and their survival is maintained by a weak signal (tonic signal) generated by the B cell receptor (BCR) in a PI3K-dependent manner (1, 2). After antigen stimulation, naïve B cells undergo rapid proliferation and differentiation into short-lived antibody-secreting plasmablasts (PBs). In the presence of T cell help, antigen-stimulated B cells undergo class switch recombination (CSR) and somatic hypermutation (SHM) and finally differentiate into long-lived memory B or plasma cells (PC) (3). These processes are regulated by signals through the BCR, toll-like receptors (TLR), CD40, cytokine and chemokine receptors, and costimulatory molecules such as CD80 and CD86, expressed or upregulated in B cells. PI3K is a key effector downstream of these receptors (4, 5), and is composed of three classes (Class IA, IB; II; III). Class IA PI3Ks are heterodimers containing a catalytic subunit (p110α, p110β or p110δ) and a regulatory subunit (p85α, p85β or p55). While p110α and p110β are broadly expressed, p110δ is predominately expressed by leukocytes (5, 6) and is the major isoform activated in B cells (7, 8).

Recent studies have identified mutations in the *PIK3CD* gene, which encodes the p110δ catalytic subunit, in patients with primary immunodeficiencies (9–12). Most patients present with recurrent respiratory tract infections (RRTIs), progressive airway damage (bronchiectasis), impaired antibody responses following natural infections and vaccinations, high serum IgM levels and hypogammaglobulinemia, which suggest characteristics of a humoral immune deficiency. T cells from patients with *PIK3CD* E1021K pathogenic variant showed increased activation of PI3Kδ, elevated AKT phosphorylation and increased mTOR expression before and/or after *in vitro* stimulation (9, 10). The disease caused by these *PIK3CD* gain-of-function (GOF) mutations is designated as APDS and molecules downstream of the PI3K signaling pathway have become the therapeutic target in APDS (12, 13). In fact, targeted therapy with PI3Kδ inhibitor leniolisib has normalized the circulating transitional and naïve B cells (14).

In a previous study (15), we reviewed clinical and routine immunological features of 15 APDS patients diagnosed in our center by next-generation sequencing (NGS). In the present study, we extended our previous study to an international cohort and explored the cellular mechanisms underlying the complex and seemingly contradictory B cell phenotypes in these patients. Based on the results of the present study and previous findings, we propose a model that explains the complex phenotypes of B cells and antibodies in APDS patients.

## Methods

### Patients, Human Samples and Ethical Approval

**Figure 1:**
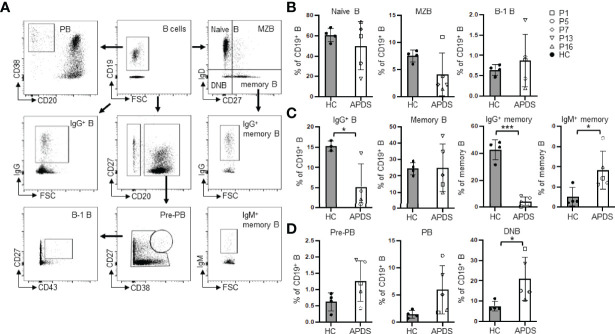
B cell subset distribution in the peripheral blood of APDS patients. **(A)** Gating strategy for the analysis of different B cell subsets. **(B)** Percentages of naïve B (CD19^+^IgD^+^CD27^-^), MZB (CD19^+^IgD^+^CD27^+^) and B-1 B (CD20^+^CD27^+^CD43^+^) cells in healthy controls (HC) and the patients (APDS). **(C)** Proportions of IgG^+^ (class-switched) B, total memory B (CD19^+^IgD^-^CD27^+^), and IgG^+^ and IgM^+^ memory B cells among the total memory B cells in HC and the patients. **(D)** Proportions of pre-plasmablast (pre-PB, CD38^hi^CD27^hi^), plasmablast (PB, CD20^-^CD38^hi^) and double negative B (DNB, CD19^+^IgD^-^CD27^-^) cells in HC and the patients. Grey column, HC (n=4); Open column, patient (n=5). *p < 0.05; ***p < 0.001 (unpaired t-test). Bars indicate mean ± SD.

**Figure 2:**
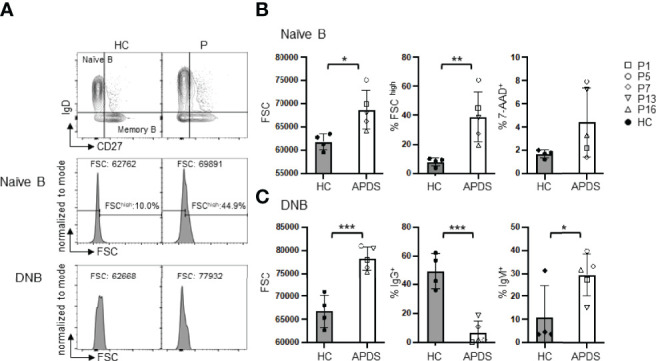
Both naïve B and DNB cells from the patients have larger sizes than those from HC. **(A)** Representative FACS profiles of FSC in gated naïve B and DNB cells. **(B)** FSC (left), % FSC^high^ (middle) and % 7-AAD^+^ (right) cells in naïve B cells of HC and the patients. **(C)** Left, FSC of DNB in HC and the patients. Middle and right, proportions of IgG^+^ and IgM^+^ cells among the total DNB cells. Grey column, HC (n=4); Open column, patient (n=5). *p < 0.05; **p < 0.01; ***p < 0.001 (unpaired t-test). Mean ± SD is shown.

**Figure 3:**
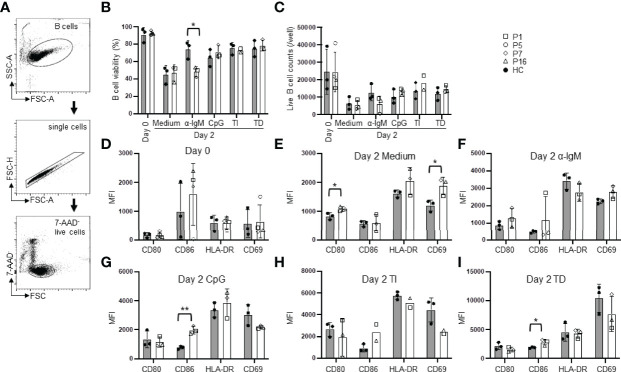
B cells from the patients show reduced survival and altered activation. Purified naïve B cells were cultured for 2 days in medium alone or in the presence of the indicated stimuli and analyzed for B cell survival and activation. **(A)** Gating strategy for live cells. **(B)** B cell viability (%) before (day 0) and after culture (day 2) with medium alone or α-IgM Abs (BCR signal), CpG2006 (TLR9 signal), α-IgM + CpG + IL-2 (T cell-independent stimuli, TI), or SA-CD40L plus IL-4 (T cell-dependent stimuli, TD). **(C)** Live B cell numbers before (day 0) and after culture. **(D–I)** Expression levels (mean fluorescence intensity, MFI) of CD80, CD86, HLA-DR, CD69 on freshly isolated B cells **(D)** or B cells cultured in medium alone **(E)** or with the indicated stimuli **(F–I)** for 2 days. Grey column, HC (n=3-4); Open column, patient (n=3-5). Mean ± SD is shown. *p < 0.05; **p < 0.01 (unpaired t-test).

**Figure 4:**
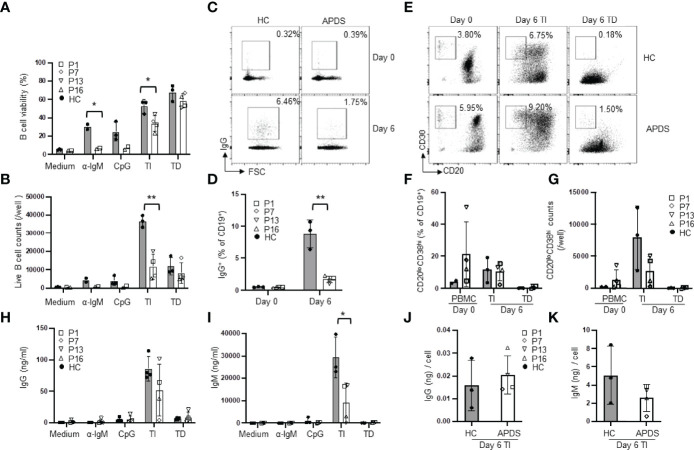
Reduced CSR but relatively normal plasma cell differentiation in patients’ B cells. Purified naïve B cells were cultured for 6 days in medium alone or in the presence of the indicated stimuli and analyzed for B cell survival, Ig gene CSR, plasma cell differentiation and antibody secretion. **(A)** B cell viability. **(B)** Live B cell numbers. **(C, D)** The proportions of IgG^+^ B cells in freshly isolated naïve B (day 0) and naïve B cells cultured for 6 days with CD40L + IL-4. Representative FACS profiles **(C)** and results of 3 HC and 4 patients **(D)** are shown. **(E–G)** *In vitro* PB differentiation. **(E)** Representative FACS profiles of CD19^+^ cells in PBMC (day 0) and in naïve B cells cultured for 6 days with TI or TD stimuli. **(F)** Proportion and **(G)** number of plasmablasts in HC and the patients. (H and I) Naïve B cells were cultured under the indicated conditions for 6 days and the levels of IgG **(H)** and IgM **(I)** in the culture supernatants were measured by ELISA. **(J, K)** IgG **(J)** and IgM **(K)** secretion on a per PB basis. The amount of IgG **(H)** or IgM **(I)** in the culture supernatants of TI stimuli was divided by the total number of PB shown in **(G)**. HC, n=2-3; Patient, n=2-4. *p < 0.05; **p < 0.01 (unpaired t-test).

**Figure 5:**
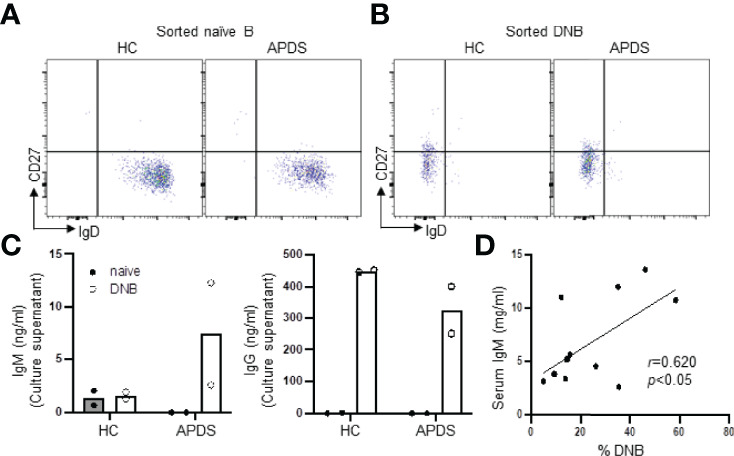
DNB cells from a patient with APDS produced both IgM and IgG upon stimulation. Naïve B and DNB cells were sorted from PBMC of a HC and a PIK3CD patient (P13) and cultured in the presence of R848, BAFF, IL-21, IL-2 and IFN-γ for 4 days. IgM and IgG levels in the culture supernatants were then measured by ELISA. (A and B) FACS profiles of sorted naïve **(A)** and DNB **(B)** cells. **(C)** Levels of IgM (left) and IgG (right) Abs in the culture supernatants of naïve B vs. DNB cells from a HC and a patient. Data from duplicate wells are shown. **(D)** Spearman’s correlation analysis revealed a strong association between the percentage of DNB cells and serum IgM levels in APDS patients (n=11). Spearman’s rank correlation coefficient *r*- and *p*-values are shown.

**Figure 6:**
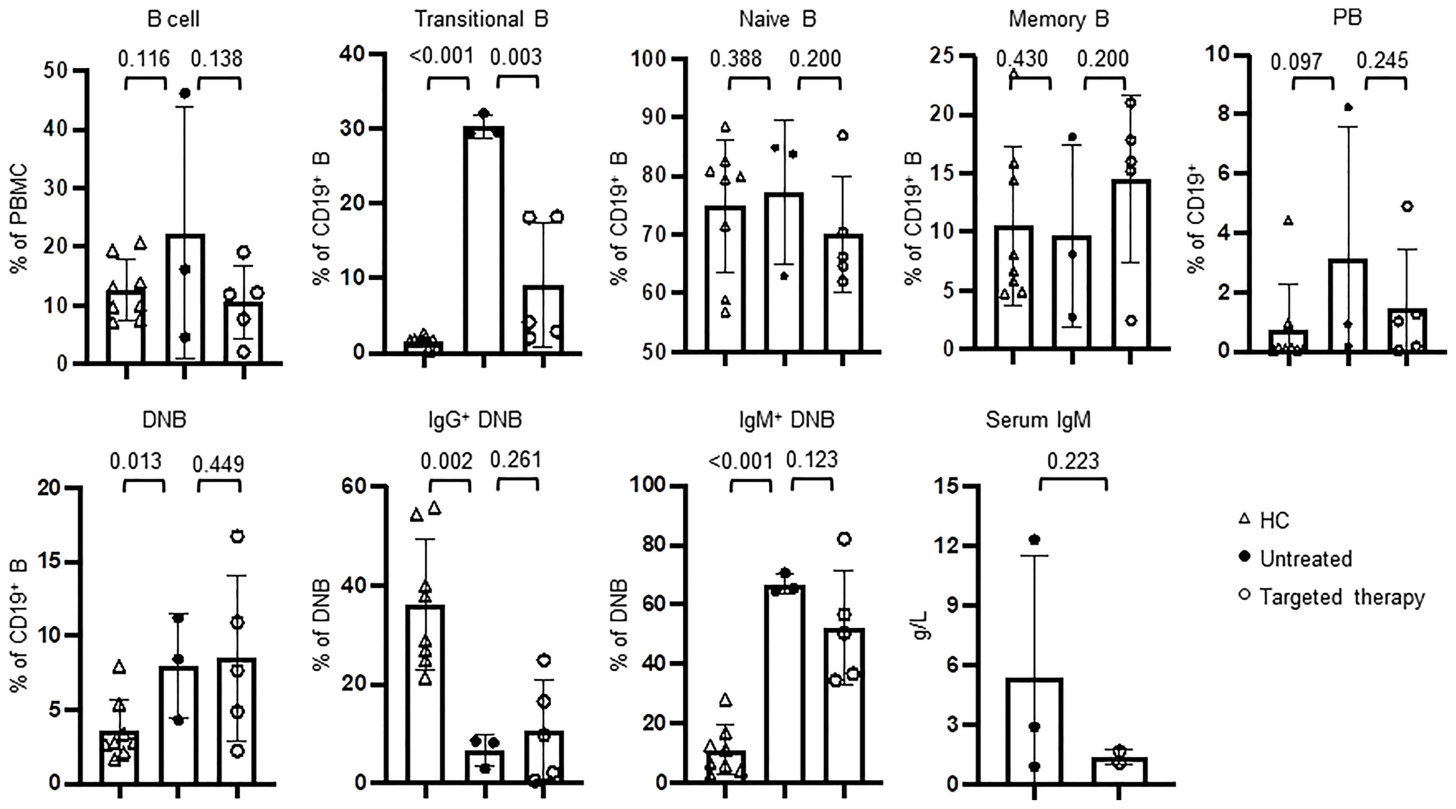
Targeted therapy to PI3K pathway ameliorated the abnormal B cell phenotypes in APDS patients. The peripheral blood from HC (n=8), the patients who had not been treated (n=3) and those who received targeted therapy (n=5) was analyzed for the percentages of different B cells subsets by flow cytometry and serum IgM by ELISA. P values were calculated by one-tailed unpaired t-test.

Clinical and laboratory data of patients and peripheral blood samples of APDS patients were obtained from Children’s Hospital of Fudan University (P1-P17) and University of South Florida (USF) and Johns Hopkins All Children’s Hospital (JHACH) at St. Petersburg of Florida (P18-P24). Patients 1, 5, 7, 13 and 16 were analyzed for B cell subset distribution and B cell survival, activation and differentiation (**Figures 1**–**5** and **Supplemental Figure 1**). P18-P24 were analyzed for the effects of the targeted therapy on B cell subset changes and serum IgM levels (**Figure 6** and **Supplemental Tables 1** and **2**). Approval for this study was obtained from the human research ethics committee of Children’s Hospital of Fudan University (2019–048) and USF/JHACH [USF-Pro00025693 (PI: J.W.), JHMI-IRB00175372 (PI: J.W.)]. All participants of this research have signed an informed consent.

### Human Antibodies and Flow Cytometry

Single-cell suspensions from peripheral blood were incubated with an Fc block™ (BD Biosciences) first to block Fcγ receptor and then stained with the following Abs: APC-H7-anti-CD3 (clone SK7, BD Biosciences), FITC-anti-CD4 (clone RPA-T4, BD Bioscience), APC-anti-CD8a (clone RPA-T8, Biolegend), anti-CD45RA (clone HI100, BD Bioscience), anti-TCR αβ (clone T10B9.1A-31, BD Bioscience), anti-TCRγδ (clone B1, BD Bioscience), APC-anti-CD19 (clone HIB19, Biolegend), FITC-anti-CD20 (clone 2H7, Biolegend), APC/Cy7-anti-CD27 (clone O323, Biolegend), PE-anti-IgD (clone IA6-2, BD Biosciences), FITC-anti-IgM (clone MHM-88, Biolegend), PE/Cy7-anti-IgG (clone G18-145, BD Biosciences), PE-anti-CD43 (clone CD43-10G7, Biolegend), PE/Cy7-anti-CD38 (clone HIT2, Biolegend), CD27-V450 (clone M-T271, BD Biosciences), IgD-BV510 (clone IA6-2, BD Biosciences), FITC-anti-CD80 (clone L307.4, BD Biosciences), PE-anti-CD86 (clone IT2.2, BD Biosciences), PE-anti-HLA-DR (clone G46-6, BD Biosciences), PE-Cy7-anti-CD69 (clone FN50, BD Bioscience) and PE-anti-CD40L (clone 24-31, Biolegend). The proportion of CD3^+^CD4^+^ (CD4^+^ T), CD3^+^CD4^+^CD45RA^+^CD27^+^ (naïve CD4^+^ T), CD3^+^CD4^+^CD45RA^-^CD27^+^ (Central memory CD4^+^ T), CD3^+^CD4^+^CD45RA^-^CD27^-^ (Effector memory CD4^+^ T), CD3^+^CD8^+^ (CD8^+^T), CD3^+^CD8^+^CD45RA^+^CD27^+^ (naïve CD8^+^ T), CD3^+^CD8^+^CD45RA^-^CD27^+^ (Central memory CD8^+^ T), CD3^+^CD8^+^CD45RA^-^CD27^-^ (Effector memory CD8^+^ T), CD3^+^CD8^+^CD45RA^+^CD27^-^ (Terminal effector memory CD8^+^ T), CD3^+^CD4^-^CD8^-^ (double negative T, DNT), CD19^+^ (B), CD19^+^IgD^+^CD27^-^ (naïve B), CD19^+^IgD^+^CD27^+^ (MZB), CD19^+^IgD^-^CD27^+^ (memory B), IgG^+^ switched memory B, IgM^+^ non-switched memory B, CD19^+^IgD^-^CD27^-^ (DNB), IgG^+^ DNB, IgM^+^ DNB, CD19^+^CD20^+^CD38^hi^CD27^hi^ (pre-PB), CD19^+^CD20^+^CD27^+^CD43^+^ (B-1) B and CD20^-^CD38^hi^ (PB) cells was analyzed by flow cytometry (Becton Dickinson, Franklin Lakes, NJ, USA) using FlowJo software.

### Isolation and *In Vitro* Culture of Human Naïve B Cells

Naïve B cells were isolated from freshly isolated peripheral blood using MojoSort Human Naïve B Cell Isolation Kit (Biolegend) together with biotinylated anti-human IgG beads to remove IgG^+^ cells. Purified naïve B cells were cultured in RPMI 1640 containing 5×10^−5^ M 2-mercaptoethanol, 2 mM L-glutamine, 100 U/ml penicillin and 100 µg/ml streptomycin, supplemented with 10% heat-inactivated fetal bovine serum (all from Thermo Fisher Scientific). F(ab’)_2_ anti-human IgM Ab (α-IgM, 2.5 μg/ml; Southern Biotech), CpG ODN 2006 (CpG, 0.6 μM; Sangon Biotech), recombinant human IL-2 (IL-2, 10 ng/ml; R&D), IL-4 (20 ng/ml; R&D) and SA-CD40L (0.2 μg/ml) were freshly added. The SA-CD40L is a tetrameric form of human CD40L generated in house (16). Human naïve B cells were seeded at 1.5×10^5^ cells/ml density in 96-well plates in a final volume of 0.2 ml. All the cells were cultured at 37°C in humidified air and 5% CO_2_. Naïve B cells were cultured for two days for survival and activation assays, and six days for CSR and antibody secretion assays. For the six-day culture, half of the medium was removed on day 3 and replaced with fresh medium supplemented with SA-CD40L, α-IgM, CpG, IL-2 or IL-4.

B cell viability was determined by 7-amino-actinomycin D (7-AAD, Biolegend) staining followed by flow cytometry analysis. B cell activation was determined by their expression of costimulatory molecules including CD80, CD86, HLA-DR and CD69, and by analyzing their forward scatter (FSC), which represents cell sizes. Ig gene CSR was assessed by determining the frequency of IgG^+^ cells. Differentiation of naïve B cells to PB was assessed by their acquisition of the CD38^hi^CD20^-^ phenotype.

### Isolation of DNB and Naïve B Cells and *In Vitro* Plasma Cell Differentiation

FACS-sorted naive B and DN B cells were cultured in RPMI 1640 (Gibco, Thermo Fisher Scientific) supplemented with 10% heat-inactivated fetal bovine serum, antibiotics (100 U/mL penicillin and 100 μg/mL streptomycin), and 5×10^−5^ M 2-mercaptoethanol and supplied with TLR7 agonist R848 (1 μg/mL, *Invivo*gen), BAFF (10 ng/mL, R&D Systems, Bio-Techne), IL-21 (10 ng/mL, R&D Systems, Bio-Techne), IL-2 (50 ng/mL, R&D Systems, Bio-Techne), IFN-γ (20 ng/mL, Peprotech) and goat F(ab′)_2_ anti-human IgM (10 μg/mL, SouthernBiotech). Four days later, the supernatants were collected and the quantity of IgM and IgG Abs was analyzed by ELISA as described (17).

### ELISA

IgM and IgG levels in the culture supernatants were measured by standard ELISA as described (17). Briefly, 96-well ELISA plates (Costar) were coated with goat anti-human Ig (Cat#2010-01, Southern Biotech), blocked with 1% BSA in blocking buffer and incubated with serially diluted culture supernatants and standards. After washing, the biotin-conjugated anti-human IgG (Cat#2040-08, SouthernBiotech) or anti-human IgM biotin (Cat#2020-08, Southern Biotech) was added, followed by Avidin-HRP (Lot: B152022, Biolegend). The HRP-activity was visualized with TMB substrate reagents (Invitrogen) and OD_450_-OD_570_ was measured by spectrophotometer (Bio-Rad). The amount of Ig was calculated according to the standard curve.

### Statistical Analysis

Data obtained from flow cytometry were analyzed with FlowJo 7.6 Software (Treestar, Woodburn, OR), and exported to Excel spreadsheets. Results are presented as mean ± standard deviation (SD). Data were analyzed using t-test or linear regression test (*P* < 0.05 was considered statistically significant) by GraphPad Prism program (GraphPad Software, San Diego, CA, USA). *P < 0.05; **P < 0.01; ***P < 0.001.

## Results

### Routine Immunological Phenotypes of the Patients

A total of 24 APDS patients were studied (**Table 1**). Twenty-one patients carried the hot spot E1021K (c.3061G>A) mutation, and three patients, P13, P7 and P23, carried the previously described E1025G (c.3074A>G), Y524N (c.1570T>A) and E81K (c.241G>A) mutations, respectively. All of these missense mutations were described as GOF in previous studies (10, 18, 19). B cell counts were decreased in 20 patients (83%) whereas serum IgM levels were higher than the upper limit of the normal range in 20 patients (87%) (**Table 1**). We have recently established the reference ranges of extended T/B cell subsets in Chinese children based on age and gender (20). In 21 out of 24 patients, fresh peripheral blood was available for lymphocyte subpopulation typing (**Table 2**). Reduced proportions of naïve CD4^+^ T cells and naïve CD8^+^ T cells were observed in most of the patients. More than half of the patients showed relatively increased proportions of CD4^+^ (n=15, 75%) and CD8^+^ (n=16, 80%) effector memory T cells. The frequency of memory B cells was decreased in 9 of 24 (38%) patients. The proportions of transitional B cells were increased in 19 of 21 (90%) patients and of PB cells were increased in 12 of 19 (63%) whereas the proportions of naive B cells were decreased in 11 of 21 (52%) patients (**Table 2**).

**Table 1:** Routine immunological features in APDS patients.

Patient number	Age(y)	Diagnosed age (y)	Gender	Mutation	WBC(×10^9^/L)	T cells counts(×10^9^/L)	B cells counts(×10^9^/L)	NK cells count(×10^9^/L)	CD4/CD8	IgG(g/L)	IgA(g/L)	IgM(g/L)	target therapy, period of treatment
1	20	14	M	E1021K	10.9	1.09↓	0.17↓	2.80↑	0.75↓	1.55↓	0.99	15.3↑	rapamycin, 6yrs
2	18*	15	F	E1021K	7.7	0.82↓	0.05↓	0.55↑	0.84↓	4.95↓	2.53	43.2↑	rapamycin, 11m
3	11*	12	M	E1021K	6.9	1.75	0.09↓	0.36	0.64↓	5.26↓	6.05↑	10.4↑	no
4	19	8	M	E1021K	3.3↓	0.62↓	0.03↓	1.46↑	0.45↓	1.54↓	UD↓	3.27↑	no
5	8	3	M	E1021K	19.7↑	8.43↑	1.00	1.96↑	0.27↓	1.38↓	0.54	2.47↑	rapamycin, 5yrs
6	9	4	M	E1021K	10.4	1.30↓	0.19↓	0.19↓	0.44↓	17.15	1.43	3.77↑	rapamycin, 3yrs
7	11	7	F	Y524N	6.5	1.05↓	0.15↓	0.86↑	0.93↓	11.60	1.32	3.81↑	rapamycin, 2yrs
8	4	0	M	E1021K	6.8	1.39↓	0.90	0.28	1.43↓	5.26	0.15	1.77↑	no
9	9	4	F	E1021K	3.8↓	0.75↓	0.04↓	0.11↓	0.46↓	4.70↓	0.29↓	10.6↑	rapamycin, 5yrs
10	8	4	M	E1021K	5.0	0.69↓	0.15↓	0.40	0.66↓	17.40	1.21	2.60↑	rapamycin, 4yrs
11	14	10	M	E1021K	3.3↓	0.33↓	0.04↓	0.07↓	0.61↓	3.95↓	1.00	5.60↑	rapamycin, 2yrs
12	8*	6	M	E1021K	6.8	3.23↑	0.11↓	0.15↓	0.14↓	16.50	0.07↓	5.21↑	rapamycin, 6m
13	11	8	M	E1025G	5.1	0.83↓	0.07↓	0.36	0.30↓	9.30^#^	0.99	3.71↑	rapamycin, 3yrs
14	8	5	M	E1021K	7.7	1.25↓	0.09↓	0.10↓	0.35↓	UD↓	UD↓	5.10↑	rapamycin, 4yrs
15	7	4	F	E1021K	7.1	0.97↓	0.26↓	0.28↓	0.52↓	9.20	0.95	3.10↑	no
16	7	5	M	E1021K	7.5	1.16↓	0.18↓	0.28↓	0.84↓	10.60	0.54	3.34↑	rapamycin, 3yrs
17	17	11	M	E1021K	8.3	3.10↑	0.04↓	0.36	0.30↓	5.70↓	0.46↓	3.43↑	rapamycin, 3yrs
18	23	15	F	E1021K	n.a.	1.05↓	0.56	0.37	0.85↓	14.29^#^	1.65	1.12	leniolisib, 4yrs
19	13	11	M	E1021K	7.3	1.90	0.21↓	0.34	0.79↓	n.a.	n.a.	n.a.	no
20	10	8	F	E1021K	4.7	2.10	0.12↓	0.29↓	0.70↓	20.1↑	0.44↓	1.66	rapamycin, 2yrs
21	37	35	F	E1021K	7.2	0.65↓	0.06↓	0.12↓	1.36	20.8↑	UD↓	0.91	rapamycin, 2yrs
22	16	11	F	E1021K	3.8↓	0.75↓	0.12↓	0.11↓	1.00↓	33.1^#^	2.06	3.90↑	no
23	9	7	M	E81K	3.3↓	0.28↓	0.36	0.02↓	2.49↑	20.3↑	3.65↑	2.24↑	no
24	19	14	M	E1021K	3.4↓	0.33↓	0.14↓	0.14	0.52↓	3.63	UD	7.99↑	rapamycin, 4yrs

* Patient 2, 3, 12 died at 18,11,8 years old, respectively.

Patients 16-22 and 24, previously unpublished cases.

# After IVIG replacement.

UD, undetectable.

n.a., not available. ↓ Below the reference range. ↑ Above the reference range.

**Table 2:** Lymphocyte subpopulations in APDS patients.

P #	CD19	Transitional B	Naive B	Memory B	Plasmablsts	CD3	CD4	CD4 Naive	CD4 CM	CD4 EM	CD8	CD8 Naive	CD8 CM	CD8 EM	CD8 TEMRA	DNT
P1	5.9↓	50.8↑	71.9	10.7	8.5↑	47.5↓	52.4↑	10.3↓	48.7	41.0↑	41.1↑	17.0↓	32.7↑	28.1↑	22.1	0.72
P2	2.5↓	3.9↓	5.7↓	31.3↑	23.9↑	73.9	28.7	9.3↓	28.7↓	34.1↑	57.7↑	20.2↓	33.3	34.0↑	12.5	0.29↓
P4	2.6↓	18.8↑	48.5↓	1.3↓	20.0↑	27.3↓	23.1	4.2↓	13.0↓	67.3↑	61.2↑	4.1↓	9.6↓	32.4↑	53.9↑	0.08↓
P5	20.8↑	42.4↑	66.5	4.6↓	19.0↑	65.9	32.1	14.6↓	63.9↑	21.4↑	45.9↑	5.5↓	23.2	31.5↑	39.8↑	0.45
P6	6.6↓	73.7↑	63.4↓	15.0↑	15.5↑	75.6↑	21.6↓	3.1↓	84.5↑	12.4↑	72.6↑	20.4↓	57.7↑	12.5	9.4	0.63
P7	5.1↓	72.9↑	31.4↓	13.3	17.7↑	49.0↓	46.6↑	16.6↓	72.9↑	10.4↑	46.7↑	15.1↓	47.1↑	20.8↑	17.0	0.41
P8	28.7	72.6↑	0.1↓	2.3	1.2	54.5	51.8	52.1↓	36.0↑	11.0↑	42.8↑	20.8↓	30.3↑	30.7↑	18.3↑	0.47
P9	5.8↓	23.4↑	40.5↓	6.1↓	9.6	65.3	36.1	11.3↓	53.5↑	35.0↑	45.4↑	6.0↓	19.7	35.2↑	39.1↑	0.43
P10	16.9	75.4↑	48.7	6.8↓	6.2	56.0↓	44.0↑	17.1↓	77.1↑	5.4	51.1↑	14.5↓	60.0↑	14.7↑	10.8	0.56
P11	3.4↓	1.8↓	42.9↓	10.1	22.3↑	64.6	34.9	5.9↓	43.9	50.1↑	58.4↑	7.4↓	31.9	33.0↑	27.7↑	0.63↓
P12	3.9↓	66.1↑	77.8↑	3.3↓	9.6↑	87.7↑	14.0↓	18.4↓	71.8↑	8.6	82.1↑	10.9↓	16.2	31.9↑	40.9↑	0.16↓
P13	2.0↓	53.7↑	29.8↓	7.8	10.6↑	66.8	18.2↓	15.1↓	72.4↑	12.5↑	74.9↑	10.6↓	64.8↑	17.9↑	6.7	0.95
P14	3.3↓	71.3↑	77.4↑	4.3↓	9.1↑	83.8↑	21.3↓	12.2↓	46.7↑	35.3↑	61.8↑	5.2↓	23.7	42.7↑	28.4↑	0.51
P15	9.2↓	46.5↑	78.7↑	12.0	17.1↑	73.6	19.2↓	25.2↓	63.0↑	11.5↑	75.5↑	9.3↓	9.7↓	39.4↑	41.7↑	0.76
P16	6.8↓	52.5↑	79.2↑	3.5↓	5.4	50.2↓	30.5	19.1↓	34.0	43.2↑	42.4↑	14.3↓	35.5↑	20.8↑	29.4↑	0.27
P19	11.0	28.0↑	82.0↑	12.0	3.0	69.0	30.0	30.0	n.a.	n.a.	38.1↑	15.0↓	n.a.	n.a.	n.a.	n.a.
P20	13.0	5.0	64.0	27.0↑	6.2	77.0↑	41.0	32.0	52.0↑	10.0↑	40.0↑	38.0↓	57.0↑	5.0	0↓	n.a.
P21	9.0↓	30.0↑	68.0	22.0	5.2	70.0	55.0↑	85.0↑	10.0↓	2.0↓	39.0↑	50.0	42.0↑	3.0	1.0↓	n.a.
P22	12.5	16.0↑	49.0	17.0	21.4↑	62.0	46.0↑	12.0↓	71.0↑	16.0	46.0↑	6.0↓	76.0↑	18.0↑	1.0↓	n.a.
P23	51.0↑	2.2	33.4↓	7.4↓	n.a.	40.0↓	69.4↑	34.0↓	53.0↑	11.0	27.8	74.0↑	12.0↓	13.0	8.0	n.a.
P24	25.0↑	8.3	19.1↓	5.0↓	n.a.	59.0	29.4	9.0↓	51.0↑	39.0↑	56.4↑	58.0	11.0↓	29.0↑	29.0↑	n.a.

Lymphocyte subpopulation was not analyzed for patients 3, 17, and 18.

DNT, TCRαβ^+^ CD4 and CD8 double-negative T cell.

n.a., not available. ↓ Below the reference range. ↑ Above the reference range.

### B Cell Subset Distribution in the Peripheral Blood of APDS Patients

PBMC from 5 patients with 3 different PIK3CD mutations (patients 1, 5 and 16 with E1021K; patient 13 with E1025G; patient 7 with Y524N) and 4 HC were further analyzed for the proportions of different B cell subsets (**Figure 1**). Four patients (except patient 7) had received rapamycin treatment at the time of analysis. **Figure 1A** shows the gating strategies. No differences were observed in the proportions of naïve B (CD19^+^IgD^+^CD27^-^), MZB (CD19^+^IgD^+^CD27^+^) and B-1 B (CD20^+^CD27^+^CD43^+^) cells between the patients and HC (**Figure 1B**). The proportions of class-switched (IgG^+^) cells were significantly reduced in the patients (5.20 ± 5.63% vs. 15.30 ± 1.25% in HC) (**Figure 1C**, left panel). While the frequencies of total memory B (CD19^+^IgD^-^CD27^+^) were comparable between the patients and HC (**Figure 1C**, 2nd panel), the frequencies of IgG^+^ memory B cells (IgG^+^CD19^+^IgD^-^CD27^+^) were significantly lower in patients (4.40% ± 3.42%) compared with HC (42.60 ± 7.47%) (**Figure 1C**, 3rd panel), which is consistent with the decreased IgG^+^ cells (**Figure 1C**, left panel) and previous studies (21, 22). We found significantly higher frequencies of the IgM^+^ memory B cells (IgM^+^CD19^+^IgD^-^CD27^+^) in the patients (36.78% ± 18.90%) compared with HC (10.49 ± 9.44%) (**Figure 1C**, right panel), which had not been described previously. In agreement with a previous study (21) was the finding that the proportions of pre-PB (CD20^+^CD38^hi^CD27^hi^) and PB (CD20^-^CD38^hi^) were increased (**Figure 1D**) although the differences did not reach statistical significance. We also noted an increased proportion of the CD27^-^IgD^-^ double negative B (DNB) cells in the patients (20.92 ± 10.65% vs. 7.54 ± 2.45% in HC, *P* <.05) (**Figure 1D**, right).

### Both Naïve B and DNB Cells From APDS Patients Have Larger Sizes Than Those From HC

A previous study showed that B cells in APDS patients were in an activated state, as suggested by their elevated pAKT levels relative to B cells from HC (21). Since B cell activation is frequently associated with increased cell size, we next analyzed the sizes of naïve B and DNB cells (**Figure 2A**). Indeed, we found that naïve B cells of the patients had significantly larger cell sizes, as revealed by their increased forward scatter (FSC) and higher percentages of the FSC^high^ population than B cells from HC (**Figure 2B**, left and middle panels). Naïve B cells in the patients contained higher proportions of 7AAD^+^ dead cells than those in HC (**Figure 2B**, right panel), although the difference did not reach statistical significance. Moreover, DNB from the patients had much larger cell sizes than those from HC (*P* <.001) (**Figure 2C**, left panel). The majority of the DNB cells from the patients were IgM^+^, which was in striking contrast to those from HC that were mostly IgG^+^ (**Figure 2C**). Such DNB cells have recently been shown to be derived from activated naïve B cells and are precursors of PC (23).

### B Cells From APDS Patients Show Reduced Survival and Altered Activation

To analyze intrinsic defects in B cell function, we purified naïve B cells and analyzed their survival and activation under various *in vitro* culture conditions. We used MojoSort B cell purification kit and also included biotinylated anti-human IgG-conjugated beads to remove IgG^+^ cells. The purified B cells were mostly IgD^+^ as shown in Supplemental **Figure 1**. While freshly isolated PBMC from the patients contained an increased proportion of dead naïve B cells than those from HC (**Figure 2B**, right panel), purified naïve B cells from the patients and HC had similarly high viability (**Figure 3B**, day 0), possibly because the dead cells had been removed during the purification processes. We cultured purified naïve B cells for 2 days in medium alone or in the presence of α-IgM (BCR signal), CpG2006 (TLR9 signal), α-IgM + CpG + IL-2 (T cell-independent stimuli, TI), or SA-CD40L plus IL-4 (T cell-dependent stimuli, TD). As shown in **Figure 3B**, naïve B cells from the patients had significantly lower viability than those from HC when cultured in the presence of α-IgM Abs (48.37 ± 5.94% vs. 74.08 ± 9.98% in HC). Live cell counts were decreased in parallel but without statistically significant difference (**Figure 3C**). These observations suggest that B cells from the patients had an intrinsic defect in antigen-triggered survival. Although naïve B cells from the patients had larger sizes than those from HC (**Figure 2B**), they expressed similar levels of CD80, CD86, HLA-DR and CD69 as naïve B cells from HC (**Figure 3D**). Patients’ B cells cultured in medium alone had elevated levels of the costimulatory molecule CD80 and the early activation marker CD69 than did HC B cells (**Figure 3E**). After the stimulation, they expressed higher levels of CD86 but downmodulated CD69 expression (**Figures 3G**–**I**). These observations suggest that the patients’ B cells exhibit enhanced activation.

### B Cells From APDS Patients Had Reduced Ig Gene CSR but Relatively Normal PB Differentiation and Antibody Secretion

To analyze abnormalities in B cell differentiation into PC, we next cultured purified naïve B cells for 6 days in medium alone or in the presence of various stimuli. In medium alone, B cells from both the patients and HC died during the 6 day-culture (**Figures 4A, B**). In the presence of α-IgM, the majority of the patients’ B cells died while ~30% of the HC B cells were still alive (**Figures 4A, B**), consistent with the results of the 2-day culture (**Figure 3B**). When cultured with CpG alone or TI stimuli, patients’ B cells also showed significantly reduced viability (**Figure 4A**) and a dramatic decrease in the live cell numbers (**Figure 4B**). The viability and number of live B cells were not different between the patients and HC after 6 day-culture with TD stimuli (**Figures 4A, B**). TD stimuli are known to induce CSR. As shown in **Figures 4C, D**, the frequency of IgG^+^ cells among CD19^+^ B cells in the patients was greatly reduced compared to HC (1.69 ± 0.52% in the patients vs. 8.80 ± 2.24% in HC). These results are in agreement with a recent study (22) showing that B cells from APDS patients failed to generate IgG^+^ PB in response to CD40L + IL-21 and indicated that elevated activation of PI3K pathway inhibited Ig gene CSR in B cells.

PB are precursors of PC with shorter life span and are mainly produced by T cell-independent antigen stimulation in the blood circulation (24). Stimulation of B cells with TLR9 ligand CpG + IL-2 was reported to induce PC generation in human B cells (25, 26). We then cultured purified naïve B cells with CpG + α-IgM + IL-2 (TI stimuli) for 6 days (**Figure 4E**), which induced efficient PB differentiation accompanied by extensive B cell expansion (**Figure 4G**). We found that B cells from HC and the patients generated a similar proportion of the CD20^low^CD38^high^ PB (**Figure 4F**), suggesting that *PIK3CD* GOF did not affect naïve B cell differentiation into PB induced by TI stimuli. However, because the cell viability and the live cell counts were both reduced in the patients compared with HC (**Figures 4A, B**), the numbers of PB were lower than those generated by HC B cells (**Figure 4G**). Consistently, PB from the patients secreted lower amounts of IgG (**Figure 4H**) and IgM (**Figure 4I**) than those from HC, although the former did not reach statistical significance. We further calculated antibody secretion on a per PB basis by dividing the amount of IgG and IgM in the culture supernatants by the total number of PB shown in **Figure 4G**. We found that PB from HC and the patients secreted comparable amounts of antibodies (**Figures 4J, K**). Stimulation with CD40L + IL-4 (TD stimuli) was not as efficient as TI stimuli in inducing PB differentiation (**Figures 4E**–**G**) and B cells from HC and the patients generated similar percentages (**Figure 4F**) and numbers (**Figure 4G**) of PB. Collectively, while the patients’ B cells are impaired in CSR, they can differentiate into PB and secrete comparable levels of antibodies as HC B cells.

### DNB Cells From a Patient With APDS Secreted More IgM but Comparable IgG Compared With Those From a HC

To explore the pathogenicity of the DNB in the patients, we sorted naïve B (**Figure 5A**) and DNB (**Figure 5B**) cells from a HC and a patient (P13) and cultured them under mixed stimuli (TLR7 agonist R848 + BAFF + IL-21 + IL-2 + IFN-γ + F(ab′)_2_ anti-human IgM) for 4 days. We found that while DNB cells from the HC and the patient secreted comparable amounts of IgG (**Figure 5C**, right), only the patient’s DNB cells secreted detectable levels of IgM (**Figure 5C**, left). In contrast, naïve B cells from both the HC and the patient produced neither IgM nor IgG (**Figure 5**). These results are consistent with the higher frequency of IgM^+^ DNB cells in the patients than in the HC (**Figure 2B**, right panel). Moreover, we found that the serum IgM levels positively correlated with the percentages of the DNB cells in 13 APDS patients who were analyzed for the DNB population (**Figure 5D**).

### Targeted Therapy to PI3K Signaling Pathway Partially Restored B Cell Phenotypes in APDS Patients

Rapamycin and leniolisib are used as targeted therapeutic drugs for APDS patients. We then compared B cell phenotypes in three patients (P19, P22, P23) who had not received the treatment and five patients (P18, P20-P22, P24) who had received the treatment. Among these, one patient (P22) was analyzed before and after the treatment. Compared with HC, the untreated patients had significantly increased proportions of transitional B and IgM^+^ DNB and decreased proportion of IgG^+^ DNB cells, which were partially normalized in the patients with targeted therapy (**Figure 6**), Although statistically not significant, the increased percentage of PB and elevated levels of serum IgM were also partially ameliorated (**Figure 6**). As shown in **Supplemental Table 1**, P22 had improved AIHA with Coombs test turning negative after the treatment. Consistently, the targeted therapy partially normalized B cell phenotypes in P22, as revealed by the decreased proportions of transitional B, IgM^+^ memory B and IgM^+^ DNB populations and the increased proportions of IgG^+^ memory B and IgG^+^ DNB cells (**Supplemental Table 2**).

## Discussion

In the present study, we found that APDS patients had a significantly increased CD27^-^IgD^-^ (DNB) population compared to HC and the frequency of DNB cells positively correlated with the serum IgM levels in the patients. These DNB cells did not express IgD so they are different from activated IgM^+^IgD^+^ naïve B cells. Since we did not analyze the expression of CD11c, CD21, CXCR5 and CXCR3, it is unclear whether these DNB cells correspond to the previously identified DN2 cells (27). This DNB subset was initially found to be increased in patients with autoimmune or autoinflammatory diseases (28). Phenotypic, functional and proteomics analysis suggested that the DNB cells are derived from activated naïve B cells and are precursors of PC (23). We have recently shown that in patients with hypomorphic RAG expression, DNB cells are increased. Upon *in vitro* stimulation, such DNB cells quickly differentiated into IgG-secreting PC (17). We postulated that the B cell lymphopenia in these patients triggered homeostatic proliferation of B cells, resulting in the generation of DNB cells. In the case of APDS patients, we propose that the enhanced activation of naïve B cells, as revealed by their increased pAKT levels and pS6 expression shown previously (9, 21), as well as the upregulated expression of CD69 and increased cell size shown in the present study, may result in the generation of DNB cells. In contrast to DNB cells in hypomorphic RAG patients and in HC, DNB cells from APDS patients secreted not only IgG but also IgM upon stimulation. This result is consistent with the finding that the majority of DNB cells in APDS patients are IgM^+^.

Wentink et al. have cultured total PBMC and found that B cells of APDS patients had elevated proportions of apoptotic cells compared with HC (21). We found that naïve B cells purified from APDS patients showed increased death upon α-IgM stimulation for 2 or 6 days or CpG or TI stimulation for 6 days, further indicating that patients’ B cells were intrinsically prone to undergo cell death. Paradoxically, while patients’ B cells showed increased death, they expressed higher levels of CD86 than HC B cells after stimulation. Therefore *PIK3CD* GOF B cells showed elevated activation accompanied by increased death.

Mature B cells in the periphery depend on two central survival determinants, the BCR and the receptor for the tumor necrosis factor (TNF) family cytokine, BAFF (29, 30). It has been shown that mature B cells undergo apoptosis and disappear from the body with a half-life of 3-6 days upon *in vivo* BCR ablation or mutation of one of its signaling units, the Igα polypeptide chain (30, 31). Remarkably, mature B cells losing their BCR (BCR^neg^) were fully rescued by either transgenic expression of constitutively active PI3K or knockout of the *Pten* gene, which encodes an inhibitor of PI3K signaling (32). In both rescue cases, however, the PI3K activity levels were only modestly above those in resting BCR^+^ B cells, and much lower than the levels induced by B cell activation (32). These studies demonstrate that optimal PI3K activity is critical for BCR-triggered B cell survival and activation. The increased death of PIK3CD GOF B cells could be caused by excessive PI3K activity.

The frequency of IgG^+^ cells among total CD19^+^ B cells was significantly decreased in the patients compared with HC. In addition, CSR to IgG in patients’ B cells was impaired under *in vitro* culture conditions. These *in vivo* and *in vitro* data indicate that patients’ B cells have an intrinsic defect in CSR, in agreement with the previous studies showing that PI3K activity inhibits AID expression (33, 34). Since AID is also required for the somatic hypermutation of Ig genes and affinity maturation, APDS patients may have defects in generating high-affinity antibodies against pathogens, which would render them more susceptible to infections. Moreover, imbalanced CSR and plasma cell differentiation could also promote autoimmune conditions as observed in patients with dysregulated PTEN expression (35, 36). Our data complement previous observation that naïve B cells from APDS patients were impaired in differentiating into IgG-secreting PB after stimulation with CD40L + IL-21 (22) and more directly reveal a defect of CSR in *PIK3CD* GOF B cells. Avery et al. generated a mouse model of *PI3KCD* gain of function (E1021K) and showed that CSR efficiency of mutant B cells was reduced to approximately half of that seen for WT B cells (22). It appears that human B cells with *PIK3CD* GOF mutations have a more severe defect in CSR than the corresponding mouse B cells and, in fact, some of the patients were initially diagnosed as having hyper IgM syndrome. The majority of the memory B and DNB cells in HC are IgG^+^, suggesting that they are derived from class-switched B cells. In contrast to HC B cells, patients’ B cells are impaired in CSR and may become memory B and DNB cells without undergoing CSR, resulting in high proportions of IgM^+^ memory B and IgM^+^ DNB cells.

**Figure 7:**
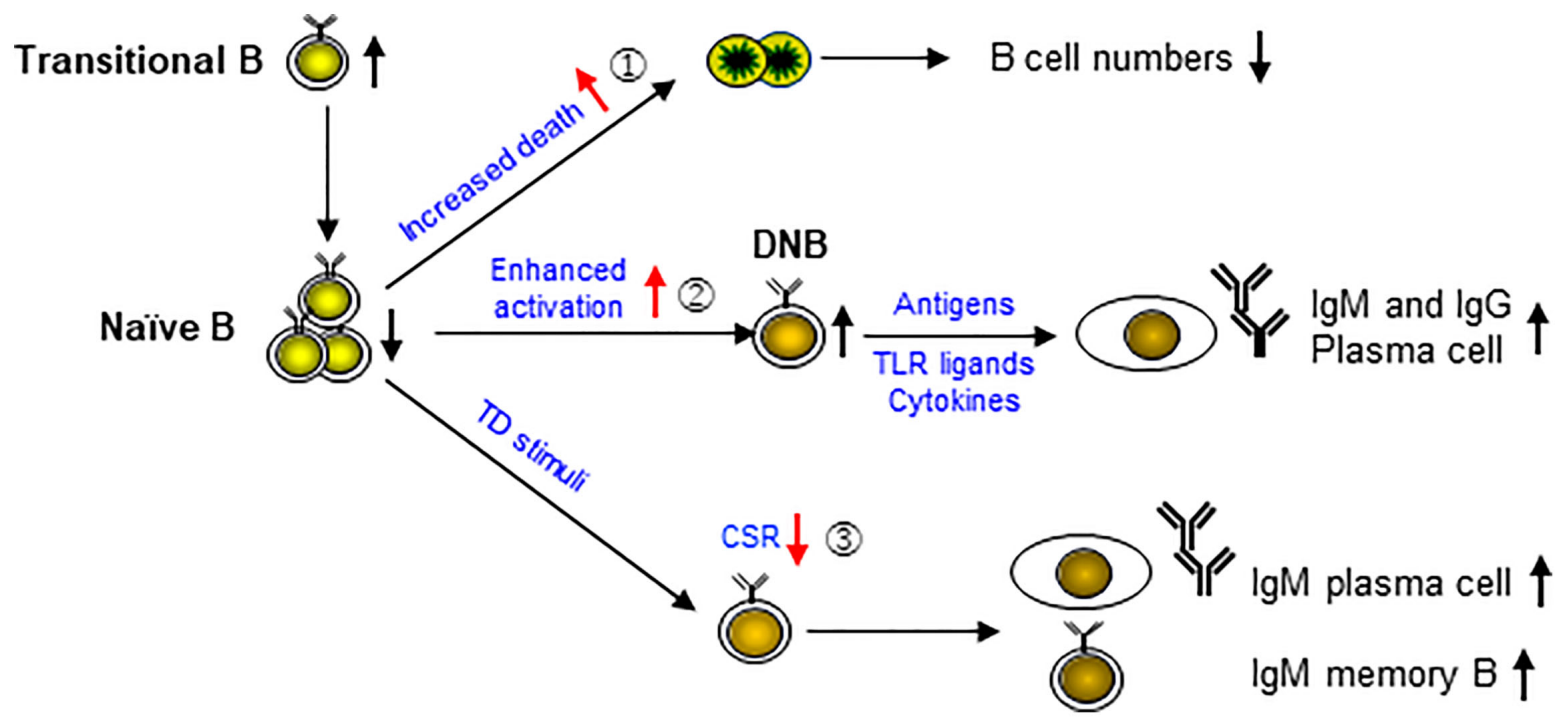
A model for cellular mechanisms underlying the complex B cell phenotypes in APDS patients. *PIK3CD* GOF has been shown to cause the accumulation of transitional B cells and a reduction of naïve B cells. Our data indicate that naïve B cells are prone to die upon stimulation (①), leading to progressive B cell lymphopenia. In addition, naïve B cells are in an activated state (②), which may result in the generation of DNB cells that can further differentiate into IgM- or IgG-secreting plasma cells upon stimulation. Moreover, upon TD stimuli, activated B cells are impaired in CSR (③) but show relatively normal plasma cell differentiation, resulting in the generation of IgM-secreting plasma cells and possibly of IgM^+^ memory B cells.

Based on the results of previous studies (9, 10, 21, 22) and the present study, we propose a model for the complex B cell phenotypes in APDS patients (**Figure 7**). *PIK3CD* GOF has been shown to cause an increase of transitional B cells in part *via* an increased output of cells from the bone marrow (21), and a reduction of naïve B cells. Our data indicate that naïve B cells are prone to die upon α-IgM, CpG or TI stimulation (①), leading to progressive B cell lymphopenia. In addition, naïve B cells show enhanced activation (②), which may result in the generation of DNB cells that can further differentiate into IgM- or IgG-secreting PC upon stimulation. Moreover, upon TD stimuli, activated B cells are impaired in CSR (③) but show relatively normal PC differentiation, resulting in the generation of IgM-secreting PC and possibly of IgM^+^ memory B cells. Our model fits very well with the complex B cell phenotypes in the patients published thus far and suggests that the key to normalize B cell function is to prevent enhanced B cell activation by restoring normal BCR signaling.

## Data Availability Statement

The original contributions presented in the study are included in the article/**Supplementary Material**. Further inquiries can be directed to the corresponding authors.

## Ethics Statement

The studies involving human participants were reviewed and approved by Children’s Hospital of Fudan University, China and University of South Florida (USF) and Johns Hopkins All Children’s Hospital (JHACH) at St. Petersburg of Florida. Written informed consent to participate in this study was provided by the participants’ legal guardian/next of kin.

## Author Contributions

WW, XW, and J-YW designed the study. WW, QM, and NL performed the experiments and analyzed data. XW, WW, JS, WY, JH, QZ, XH, and BS diagnosed, treated, and followed up the patients of the Children’s Hospital of Fudan University. YW and XM performed the lymphocyte phenotyping. KC analyzed B cell phenotypes in patients who had and had not received rapamycin treatment. BU, SG, DB, CS, MB, and JEW provided the patients’ clinical features and peripheral blood, and analyzed the data. WW and LL abstracted the information from electronic medical chart and follow-up material. WW wrote the manuscript, and JEW, XW, and J-YW reviewed and revised the manuscript. JEW, XW, and J-YW supervised the study. All the authors contributed to the article and approved the submitted version.

## Funding

This work was supported by the Major Research Plan of the National Natural Science Foundation of China (grant no. 91942302 to J-YW), the National Key R & D Plan of the Ministry of Science and Technology (grant no. 2019YFE0100600 to J-YW), the National Natural Science Foundation of China (grant no. 31870898 to J-YW), Projects of International Cooperation and Exchanges NSFC (grant no. 82011540008 to J-YW), Johns Hopkins All Children’s Hospital Institutional Grant (JEW), Jeffrey Modell Foundation (JEW), Robert A. Good Endowment, USF Foundation (JEW).

## Conflict of Interest

JEW is a consultant on APDS and leniolisib with Pharmin (Netherlands).

The remaining authors declare that the research was conducted in the absence of any commercial or financial relationships that could be construed as a potential conflict of interest.

## Publisher’s Note

All claims expressed in this article are solely those of the authors and do not necessarily represent those of their affiliated organizations, or those of the publisher, the editors and the reviewers. Any product that may be evaluated in this article, or claim that may be made by its manufacturer, is not guaranteed or endorsed by the publisher.

## Abbreviations

APDS, activated PI3K delta syndrome; BCR, B cell receptor; CSR, class switch recombination; DNB, double-negative B; HC, healthy control; GOF, gain-of-function; PB, plasmablast; PC, plasma cell; PI3K, phosphoinositide 3 kinase; PIK3CD, gene encoding the PI3K catalytic subunit δ, p110δ; TLR, toll-like receptor.
