# Supplementary material for: Cellular Mechanisms Underlying B Cell Abnormalities in Patients With Gain-of-Function Mutations in the PIK3CD Gene
Source: Front Immunol. 2022 Jun 21;13:890073. doi: 10.3389/fimmu.2022.890073 (PMC9253290; doi:10.3389/fimmu.2022.890073)
Supplement: Supplementary file 1 [file Presentation_1.pptx]

## Slide 1
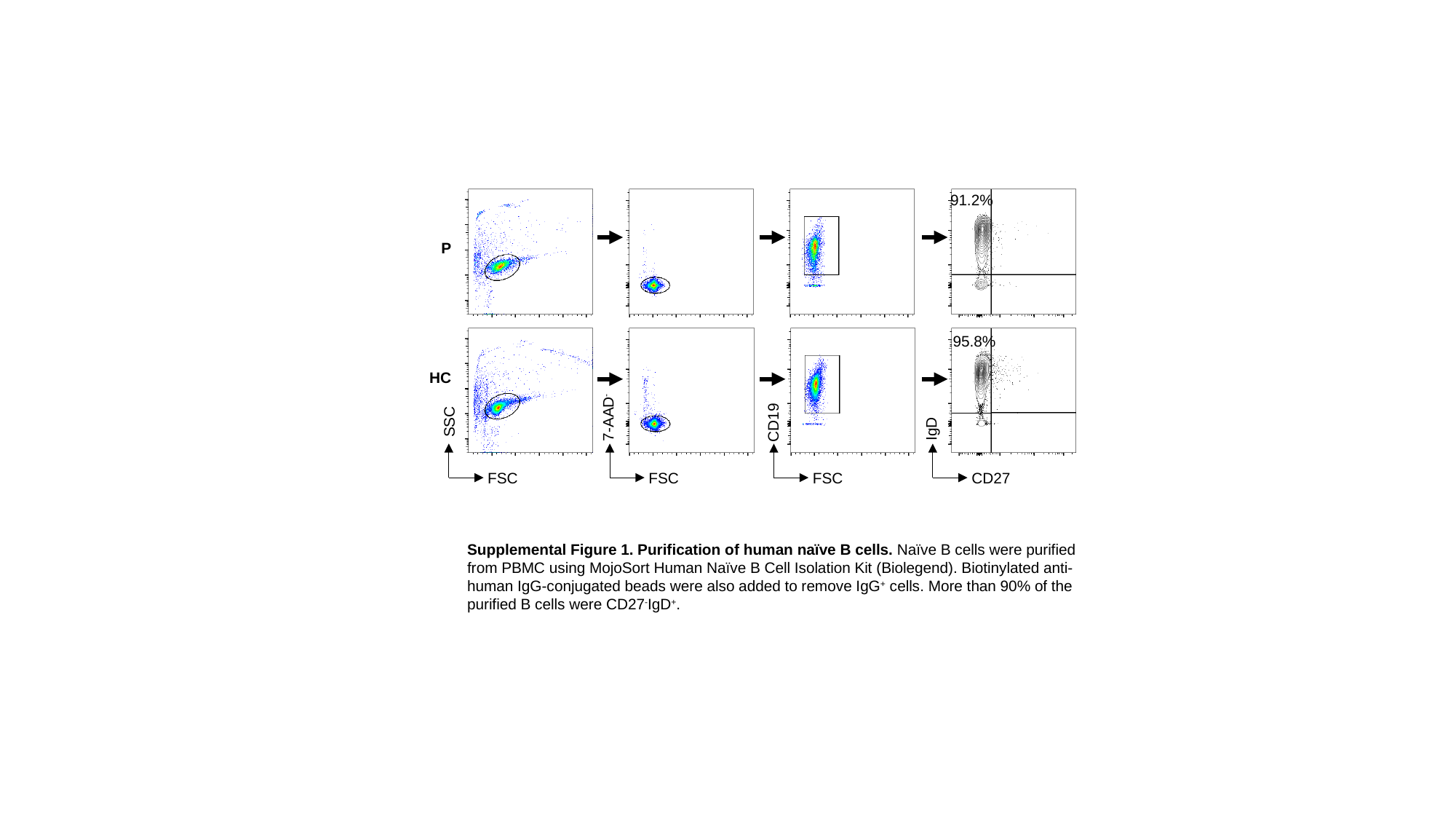

91.2%
P
95.8%
HC
7-AAD-
FSC
CD19
FSC
SSC
FSC
IgD
CD27
Supplemental Figure 1. Purification of human naïve B cells. Naïve B cells were purified from PBMC using MojoSort Human Naïve B Cell Isolation Kit (Biolegend). Biotinylated anti-human IgG-conjugated beads were also added to remove IgG+ cells. More than 90% of the purified B cells were CD27-IgD+.
