# Supplementary material for: Cellular Mechanisms Underlying B Cell Abnormalities in Patients With Gain-of-Function Mutations in the PIK3CD Gene
Source: Front Immunol. 2022 Jun 21;13:890073. doi: 10.3389/fimmu.2022.890073 (PMC9253290; doi:10.3389/fimmu.2022.890073)
Supplement: Supplementary file 2 [file Table_1.docx]

Supplemental table 1 Clinical manifestations in APDS patients with or without targeted therapy

| Targeted therapy | Patient  no. | Clinical status | Autoimmunity | Autoantibody |
| --- | --- | --- | --- | --- |
| yes | P1 | AIC, ASD | AIC | negative |
| yes | P2 | RRTIs, bronchiectasis, cellulitis, EBV viremia, mycosis, LAD, HSM, AIC, thrombocytopenia, enteropathy proteinuria, PAH, short stature; died of intestinal perforation | AIC, thrombocytopenia | positive |
| no | P3 | RRTIs, keratitis, LAD, HSM, AIC, enteropathy; died of infection | AIC, enteropathy | negative |
| no | P4 | AIC, enteropathy, LAD, splenectomy, short stature | AIC, enteropathy | negative |
| yes | P5 | RRTIs, keratitis, EBV viremia, mycosis | no | negative |
| yes | P6 | EBV viremia, HSM, development delay | enteropathy | positive |
| yes | P7 | RRTIs, EBV viremia conjunctivitis, LAD, HSM, short stature | thrombocytopenia | negative |
| no | P8 | HSM | no | n.a. |
| yes | P9 | RRTIs, parotitis, EBV viremia, mycosis, LAD, HSM, short stature | no | negative |
| yes | P10 | RRTIs, EBV viremia, enteropathy, encephalopathy, thrombocytopenia, mental retardation, hematuria | enteropathy, encephalopathy, thrombocytopenia, | positive |
| yes | P11 | RRTIs, bronchiectasis, warts, keratitis, EBV viremia, LAD, HSM, short stature, proteinuria and hematuria, hypothyroidism; successful HSCT | thrombocytopenia | negative |
| yes | P12 | RRTIs, bronchiectasis, warts, EBV viremia, mycosis, LAD, HSM, encephalopathy, short stature; died of respiratory failure | encephalopathy | positive |
| yes | P13 | EBV viremia, LAD, enteropathy | thrombocytopenia, enteropathy | positive |
| yes | P14 | bronchiectasis, EBV viremia | no | negative |
| no | P15 | RRTIs, splenomegaly | enteropathy | n.a. |
| yes | P16 | stable | enteropathy | n.a. |
| yes | P17 | warts, AIC | SLE, AIC | positive |
| yes | P18 | EBV viremia, HSM | AIHA, thrombocytopenia | positive |
| no | P19 | bronchiectasis, lichen nitidus, HSM | no | n.a. |
| yes | P20 | infected with COVID-19, severe encephalitis, HSM | no | negative |
| yes | P21 | recurrent infections, pneumonia since childhood, LAD | no | negative |
| yes | P22 | EBV viremia, AIHA, CLD and severe asthma, molluscum, tinea corporis, development delay | AIHA | positive |
| no | P23 | CMV viremia, LAD, EBV lymphadenitis, B cell lymphoma | IgA vasculitis | n.a. |
| yes | P24 | recurrent infections, bronchiectasis | n.a. | n.a. |

AIC, autoimmune cytopenia; ASD, atrial septal defect; RRTIs, recurrent respiratory tract infections; EBV, Epstein-Barr virus;

LAD, lymphadenopathy; HSM, hepatosplenomegaly; PAH, pulmonary artery hypertension; HSCT, hematopoietic stem cell

transplantation; SLE, systemic lupus erythematosus; AIHA, autoimmune hemolytic anemia; CLD, chronic lung disease; CMV,

cytomegalovirus; n.a., not available; P18-P24 were analyzed for the effects of the targeted therapy on B cell phenotypes and

serum IgM levels. P22 was analyzed before and after the targeted therapy.
