# Supplementary material for: Cellular Mechanisms Underlying B Cell Abnormalities in Patients With Gain-of-Function Mutations in the PIK3CD Gene
Source: Front Immunol. 2022 Jun 21;13:890073. doi: 10.3389/fimmu.2022.890073 (PMC9253290; doi:10.3389/fimmu.2022.890073)
Supplement: Supplementary file 3 [file Table_2.docx]

Supplemental table 2 B cell phenotypes in patient 22 before and after targeted therapy

| B cell subsets | Before therapy | After therapy (2 months) |
| --- | --- | --- |
| Transitional B | 29.56% | 18.10% |
| Naïve B | 62.90% | 62.00% |
| Memory B | 18.10% | 21.00% |
| IgG^+^ of memory B | 21.2% | 39.7% |
| IgM^+^ of memory B | 24.6% | 15.3% |
| PB | 8.23% | 4.91% |
| DNB | 11.2% | 10.9% |
| IgG^+^ of DNB | 8.26% | 24.8% |
| IgM^+^ of DNB | 65.4% | 34.5% |
